# LEVERAGING THE STRENGTH OF UNIQUE PARTNERS TO IMPROVE GERIATRIC EMERGENCY CARE IN RURAL CRITICAL ACCESS HOSPITALS

**DOI:** 10.1093/geroni/igac059.1335

**Published:** 2022-12-20

**Authors:** Scott Rodi

**Affiliations:** Dartmouth-Hitchcock Medical Center, Lebanon, New Hampshire, United States

## Abstract

Developing a level 1 accredited geriatric emergency department during the pandemic was challenging. The greater challenge has been to support rural critical access hospitals on their journey of becoming level 2 accredited geriatric emergency departments. This has been accomplished through a novel collaborative approach between the level 1 accredited emergency department, a newly established Geriatric Center of Excellence at Dartmouth-Hitchcock Health and a HRSA funded Geriatric Workforce Enhancement Program. This talk will focus on bringing together system partners and funders who have a history of success through: (a) development, testing and dissemination of tool kits and implementation guides; (b) boot camp training that includes interprofessional team training, QI and specific geriatric content training; (c) implementation support and coaching using data and QI methodology through learning collaboratives; (d) scaling successful models of evidence based geriatric care; (e) expertise in implementation science; (f) expertise in evaluation and (g) disseminating findings to achieve our goals.

